# Isolates of *Salmonella typhimurium* circumvent NLRP3 inflammasome recognition in macrophages during the chronic phase of infection

**DOI:** 10.1016/j.jbc.2021.101461

**Published:** 2021-12-02

**Authors:** David Cai, Willie June Brickey, Jenny P. Ting, Subash Sad

**Affiliations:** 1 Department of Biochemistry, Microbiology and Immunology, Faculty of Medicine, University of Ottawa, Ottawa, Ontario, Canada; 2 Department of Microbiology and Immunology, University of North Carolina at Chapel Hill, Chapel Hill, North Carolina, USA; 3 Centre for Infection, Immunity, and Inflammation (CI3), University of Ottawa, Ottawa, Ontario, Canada

**Keywords:** innate immunity, inflammation, host-pathogen interactions, BMM, bone-marrow-derived macrophage, LB, Luria–Bertani, MOI, multiplicities of infection, NLR, NOD-like receptor, PAMP, pathogen-associated molecular pattern, PRR, pattern-recognition receptor, SPI, *Salmonella* pathogenicity island, ST, *Salmonella typhimurium*, T3SS, type III secretion system, TLR, Toll-like receptor

## Abstract

Inflammasome signaling results in cell death and release of cytokines from the IL-1 family, which facilitates control over an infection. However, some pathogens such as *Salmonella typhimurium* (ST) activate various innate immune signaling pathways, including inflammasomes, yet evade these cell death mechanisms, resulting in a chronic infection. Here we investigated inflammasome signaling induced by acute and chronic isolates of ST obtained from different organs. We show that ST isolated from infected mice during the acute phase displays an increased potential to activate inflammasome signaling, which then undergoes a protracted decline during the chronic phase of infection. This decline in inflammasome signaling was associated with reduced expression of virulence factors, including flagella and the *Salmonella* pathogenicity island I genes. This reduction in cell death of macrophages induced by chronic isolates had the greatest impact on the NLRP3 inflammasome, which correlated with a reduction in caspase-1 activation. Furthermore, rapid cell death induced by Casp-1/11 by ST in macrophages limited the subsequent activation of cell death cascade proteins Casp-8, RipK1, RipK3, and MLKL to prevent the activation of alternative forms of cell death. We observed that the lack of the ability to induce cell death conferred a competitive fitness advantage to ST only during the acute phase of infection. Finally, we show that the chronic isolates displayed a significant attenuation in their ability to infect mice through the oral route. These results reveal that ST adapts during chronic infection by circumventing inflammasome recognition to promote the survival of both the host and the pathogen.

The innate immune system confers protection against a broad spectrum of pathogens through a series of germline-encoded receptors known as pattern-recognition receptors (PRRs), which recognize pathogen-associated molecular patterns (PAMPs) (1). This results in a cascade of downstream signaling mediated by transcription factors/kinases such as NF-κB and MAPK that lead to the development of an inflammatory response, which facilitates pathogen control (2). One of the most extensively studied families of PRRs are the Toll-like receptors (TLR), which typically respond to extracellular PAMPs including LPS and flagellin (3). TLR activation results in the initiation of proinflammatory signaling cascades, which may be mediated by transcription factors including NF-κB and AP-1.

In addition to PAMP-PRR interactions, the virulence factors of pathogens are recognized by cytosolic signaling platforms such as NOD-like receptors (NLRs) (2). Activation of NLRs results in the assembly of multimeric protein complexes called inflammasomes (4). Assembly and activation of inflammasomes result in the stimulation of various inflammatory caspases such as caspase-1, 8, and 11, which proteolytically process (activate) the proinflammatory cytokines IL-1β and IL-18 to their mature forms. In addition, activation of these caspases results in the cleavage of gasdermin D, which results in cell rupture by pyroptosis, a form of inflammatory cell death that is mediated through the inflammasome (5, 6). Activation of inflammasome signaling promotes control of infection (7).

Typhoid fever is a major cause of morbidity and mortality worldwide, primarily affecting high-risk populations (8, 9). *Salmonella* can disseminate systemically within the host and enter reservoirs such as the gallbladder where it can persist asymptomatically for extended periods of time (10, 11). *Salmonella enterica* serovar Typhimurium (ST) is a nontyphoidal strain of *Salmonella*. In humans, ST typically induces acute gastroenteritis (12). In contrast, infection of mice by ST induces a typhoid fever-like disease (13). Mice with a C57BL/6J genetic background carry a nonfunctional *Nramp1* (*Slc11a1*) allele, which makes them highly susceptible to *Salmonella* infection (14). In contrast, 129X1/SvJ background mice are resistant to infection as these mice possess a functional *Nramp1* gene, which facilitates the removal of divalent ions from the phagosomes, creating an inhospitable environment for *Salmonella* to survive intracellularly (15, 16). *Salmonella* may chronically persist in 129X1/SvJ mice for up to 1 year, mimicking the chronic carrier state observed in human typhoid fever infections (15, 17).

The NLRC4 inflammasome recognizes the *Salmonella* pathogenicity island (SPI)-I type III secretion system (T3SS) and flagellin, whereas the NLRP3 inflammasome responds to a distinct but unknown T3SS-independent signal (18, 19, 20, 21). Since inflammasome signaling involves rapid rupture of infected cells and release of the pyrogenic cytokine IL-1β, chronic activation of this signaling pathway can lead to significant inflammation and tissue damage. We reasoned that since *Salmonella* induces a chronic asymptomatic carrier state in hosts, the activation of inflammasome signaling and consequent tissue toxicity must be tapered. Herein we have tested this hypothesis in a mouse model of chronic ST infection, and we show that ST undergoes rapid modulation toward potent inflammasome signaling to reduce bacterial burden, followed by a protracted evolution toward reduced NLRP3 inflammasome signaling during chronic stages of infection. Despite this modulation of inflammasome signaling during the chronic course of infection, it did not offer any persistence or fitness advantage to the bacterium.

## Results

### Inflammasome signaling is dynamically modulated during the chronic ST infection

Cell death of macrophages and the associated release of IL-1β are considered as an important protective mechanism against intracellular pathogens. We therefore evaluated cell death of macrophages infected with ST and observed that this was dependent on Casp-1/11 (Fig. S1, *A* and *B*). Both wild-type and *Casp-1,11*-deficent mice on the B6 background displayed similar susceptibility with the majority of *Casp-1,11*-deficent mice succumbing to infection 1 day earlier than their wild-type counterparts (Fig. S1*C*). Inability to discern the role of Casp-1,11 in B6 mice *in vivo* may be related to the rapid fatality of wild-type mice within the first week of infection. C57BL/6J mice possess a G169D mutation in the *Nramp1* gene, which results in dysfunctional NRAMP1 and compromised control of phagosomal bacteria (22). We used B6.Nramp transgenic mice carrying a functional *Nramp1* gene on a B6 background (23) to evaluate the impact of Casp-1,11 during infection with ST. In contrast to B6 mice, B6.Nramp transgenic mice displayed enhanced survival (Fig. S1*D*) and *Casp-1,11*-deficent B6.Nramp mice displayed significantly enhanced susceptibility against ST in comparison to wild-type B6.Nramp mice (Fig. S1*D*). These results clearly reveal the protective role of Casp-1,11 in facilitating the control of ST *in vivo*, which requires expression of the functional *Nramp1* gene.

We sought to evaluate how inflammasome signaling becomes modulated during the chronic course of infection with ST. Although B6.Nramp mice displayed enhanced survival in comparison to B6 mice, B6.Nramp transgenic mice were not suitable for our studies since the survival of these mice was only extended to 20 to 30 days postinfection with ST (Fig. S1*D*). In contrast to the B6.Nramp transgenic mice, infection of 129X1/SvJ mice (which also express a functional *Nramp1* gene) with 2 × 10^2^ ST results in 100% survival of infected mice (Fig. S1*E*), and the mice harbor a chronic infection that is detectable for prolonged periods (Fig. S1*F*). Thus, the 129X1/SvJ model was ideal for evaluating the modulation of inflammasome signaling, if any, during the various stages of infection. We infected 129X1/SvJ mice intravenously with ST and sacrificed mice at 7-, 21-, and 120-days postinfection. The spleen, gallbladder, mesenteric lymph nodes, and inguinal lymph nodes were excised and homogenized to release the intracellular bacteria. The homogenates were immediately plated on LB agar plates supplemented with 50 μg ml^−1^ of streptomycin to determine the bacterial burden within each organ of interest. These *Salmonella* isolates were then expanded in LB broth and subsequently utilized to infect murine bone-marrow-derived macrophages (BMMs) at various MOIs to assess the ability of the isolates to induce cell death and IL-1β production.

**Figure 1:**
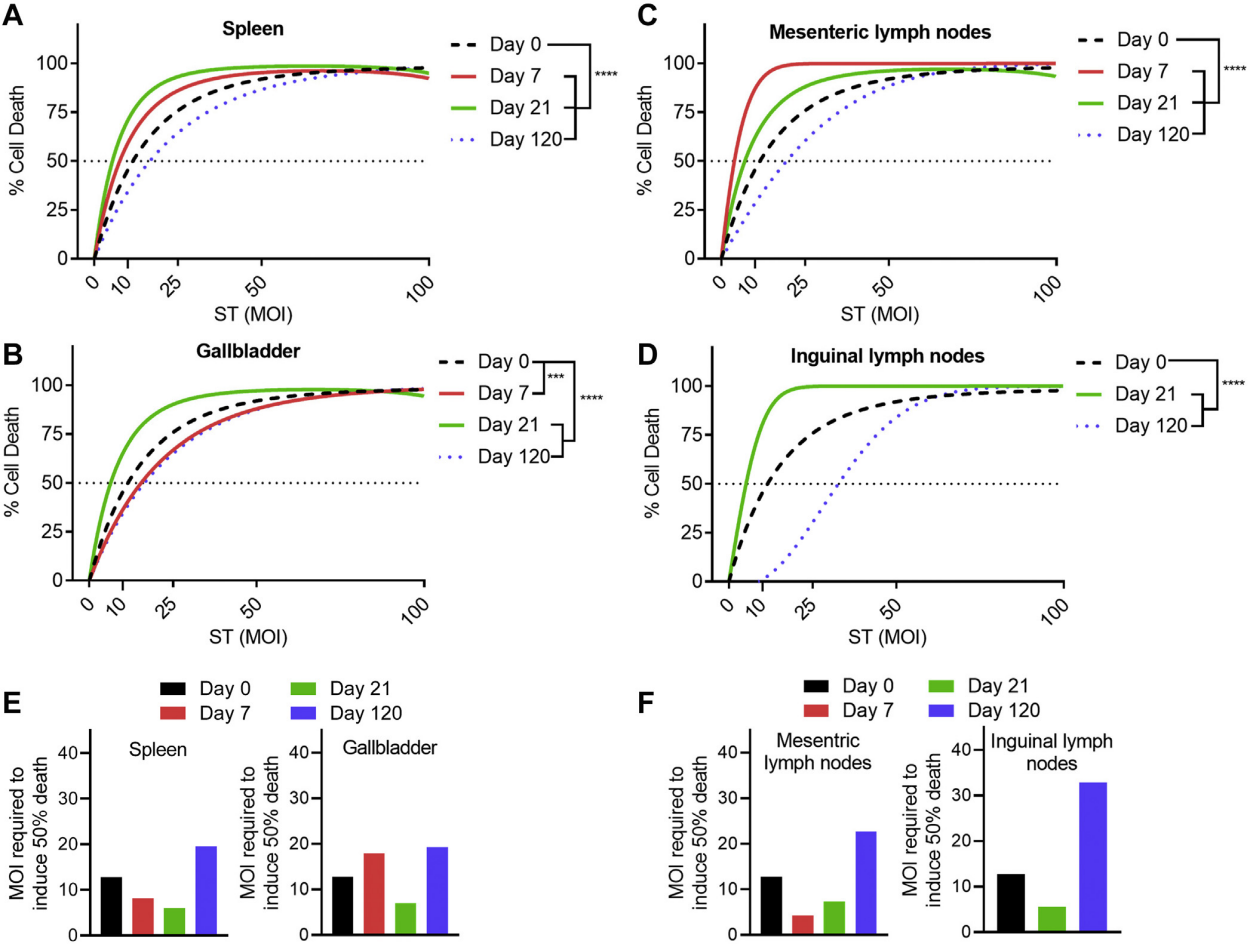
**Inflammasome signaling is dynamically modulated during chronic ST infection.** 129X1/SvJ mice were infected intravenously with 4 × 10^4^ CFU ST, and the mice were sacrificed at 7-, 21-, and 120-days postinfection. The spleen, gallbladder, mesenteric lymph nodes, and inguinal lymph nodes were excised, and serial dilutions were plated on LB agar to isolate single ST colonies. The ST isolates and ST-WT were expanded in LB broth and subsequently utilized to infect BMMs *in vitro* for 3 h. Cell death postinfection (*A*–*D*) was determined by neutral red uptake assay and is depicted using a linear quadratic survival curve fit. The MOI necessary to induce 50% cell death (*E* and *F*) was determined by LD_50_ regression in GraphPad Prism 9. Results represent the mean of nine mice at day 0, 18 mice at day 7 and 21, and 12 mice at day 120. Experiments were repeated three times. Mean values were compared by one-way ANOVA (*A*–*D*) with post-hoc Tukey’s multiple comparison test (∗∗∗*p* < 0.001; ∗∗∗∗*p* < 0.0001). BMM, bone-marrow-derived macrophage; LB, Luria–Bertani; MOI, multiplicities of infection; ST, *Salmonella typhimurium.*

**Figure 2:**
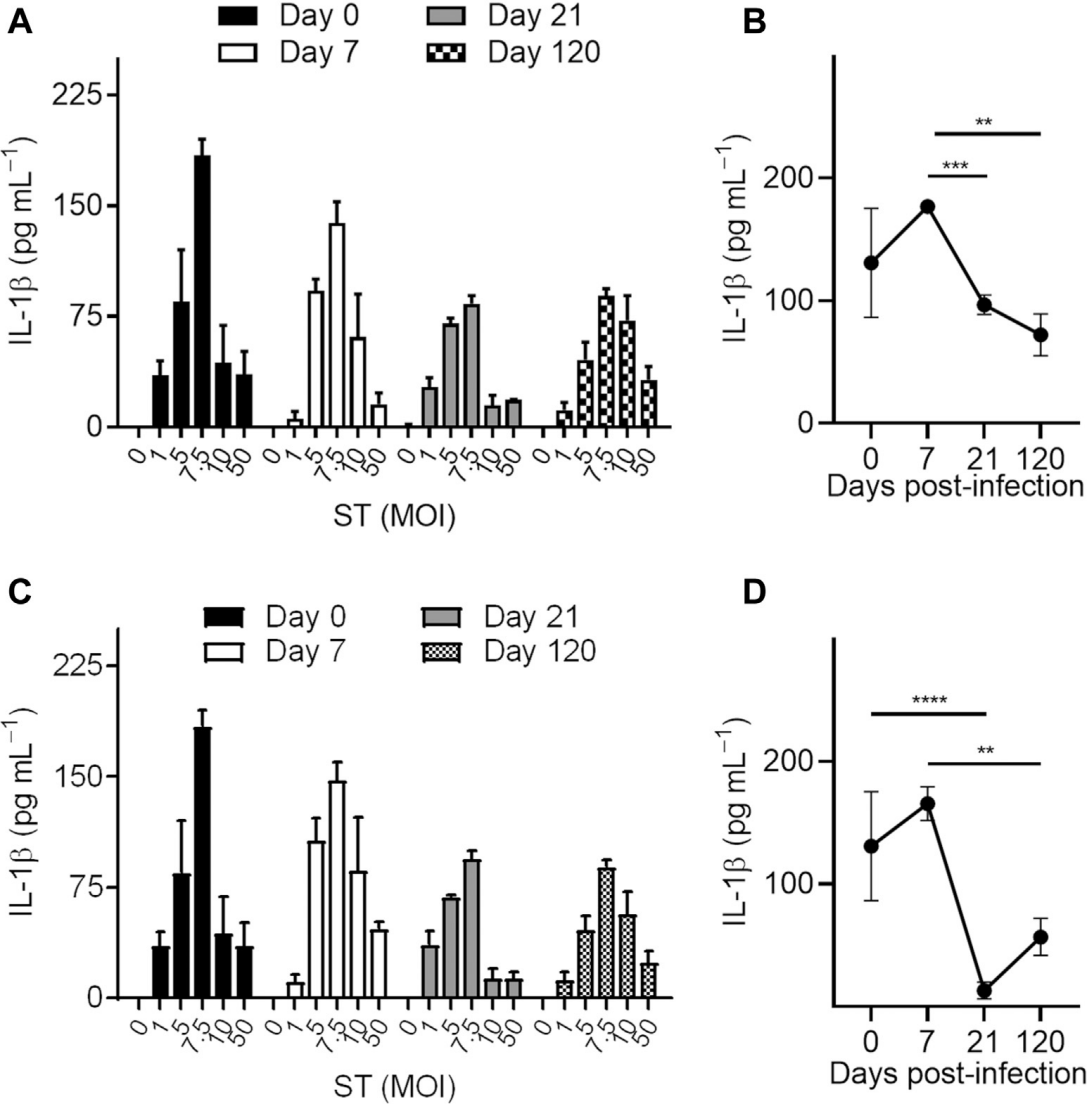
**Chronic ST isolates induce reduced IL-1β secretion.** Wild-type BMMs were infected for 3 h with ST isolates from the spleen (*A* and *B*) or gallbladder (*C* and *D*), and IL-1β secretion was quantified by ELISA. Values represent mean ± SEM. Results in panels *A* and *C* represent data pooled from five separate experiments. Results in panels *B* and *D* are representative of three separate experiments at 1 MOI. The number of mice used at each time point was the same as mentioned in Figure 1. Mean values were compared by Student’s *t* test (∗∗*p* < 0.01; ∗∗∗*p* < 0.001). BMM, bone-marrow-derived macrophage; MOI, multiplicities of infection; ST, *Salmonella typhimurium.*

We observed that there was a dynamic modulation of *Salmonella*-induced inflammatory cell death of macrophages during the various stages of infection of mice (Fig. 1). During the early stages of infection (7 d.p.i.), we observed that there was an increase in *Salmonella*-induced cell death, relative to the original *Salmonella* inoculum. This enhancement was most pronounced in bacteria isolated from the spleen and mesenteric lymph nodes (Fig. 1, *A* and *C*). At 21 d.p.i, we observed a twofold increase in the ability of the isolates to induce cell death. This marked enhancement was observed in isolates from all organs. During the late chronic stage of infection (120 d.p.i.), we observed that the ability of *Salmonella* to induce cell death was diminished, relative to the original inoculum. This effect was most significantly noted in the mesenteric and inguinal lymph nodes where we observed a two to threefold decrease in the ability of the isolates to induce cell death (Fig. 1, *C* and *D*). Modulation of cell death was more appreciable when we calculated the LD50 for cell death of macrophages (Fig. 1, *E* and *F*). During the early stages of infection, a reduced number of bacteria were required to induce 50% cell death of macrophages in comparison to the initial inoculum, whereas at the late chronic stage, an increased number of bacteria were required to achieve 50% cell death. In addition to characterizing the ability of the *Salmonella* to induce cell death, we assessed the ability of our *Salmonella* isolates from the spleen and gallbladder to induce IL-1β secretion upon infection of BMMs. Although the early isolates (7 and 21 d.p.i.) induced more cell death than the original ST-WT inoculum, we observed that all chronic isolates induced less IL-1β secretion than the original ST-WT inoculum (Fig. 2, *A*–*D*).

### Impairment in NLRP3-inflammasome signaling

**Figure 3:**
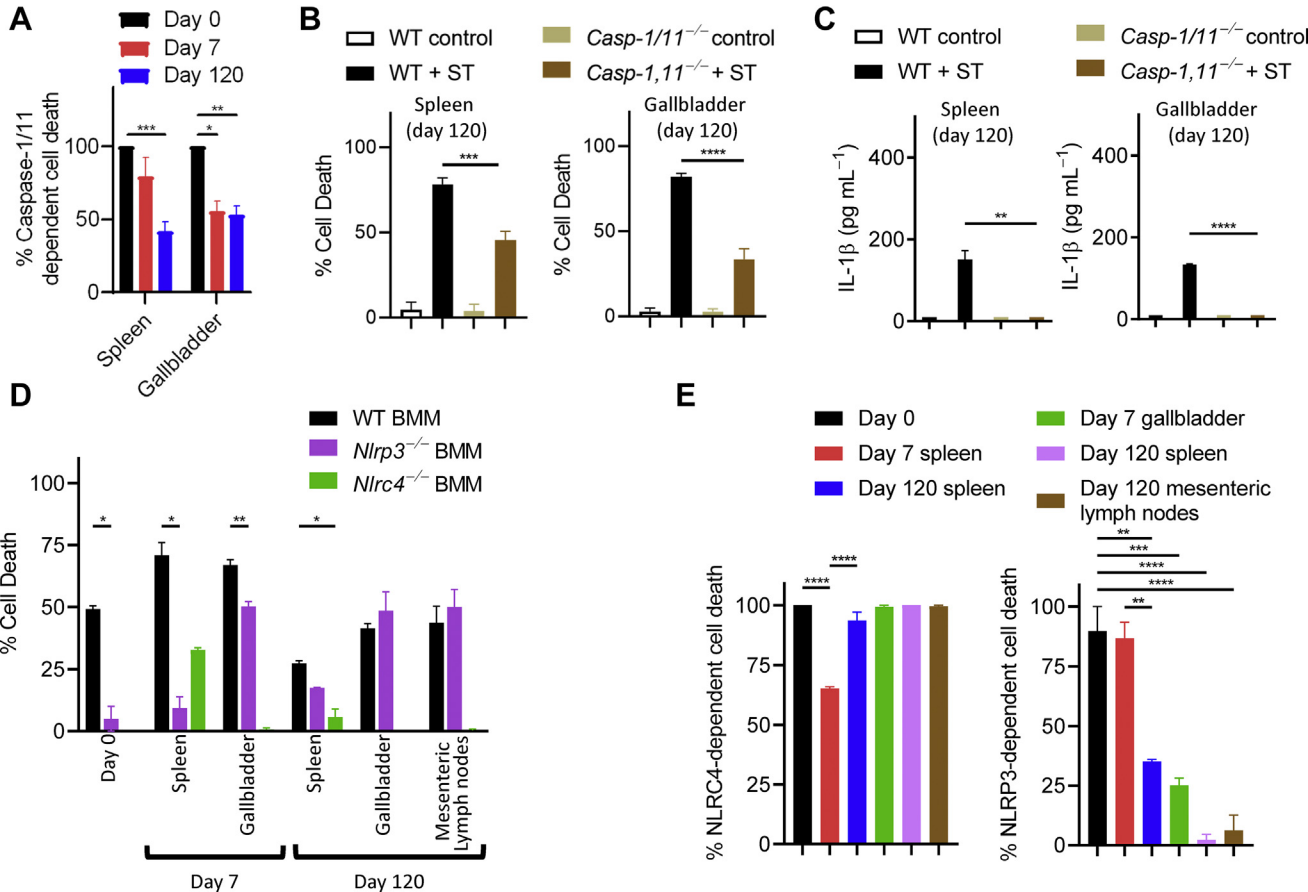
**Chronic ST isolates exhibit organ-specific modulation of inflammasome signaling.** BMMs were differentiated from mice of the indicated genotypes and infected with 10 MOI (*A*–*C*) or 1 MOI (*D* and *E*) of ST-WT or chronic ST isolates for 3 h. Cell death was determined by neutral red uptake assay. Values represent mean ± SEM of triplicate cultures. Results are representative of three separate experiments with BMMs generated from two mice/genotype in each experiment. Mean values were compared by Student’s *t* test (*A*–*C*) or one-way ANOVA (*D* and *E*) with post-hoc Tukey’s multiple comparison test (∗*p* < 0.05; ∗∗*p* < 0.01; ∗∗∗*p* < 0.001; ∗∗∗∗*p* < 0.0001). BMM, bone-marrow-derived macrophage; MOI, multiplicities of infection; ST, *Salmonella typhimurium.*

**Figure 4:**
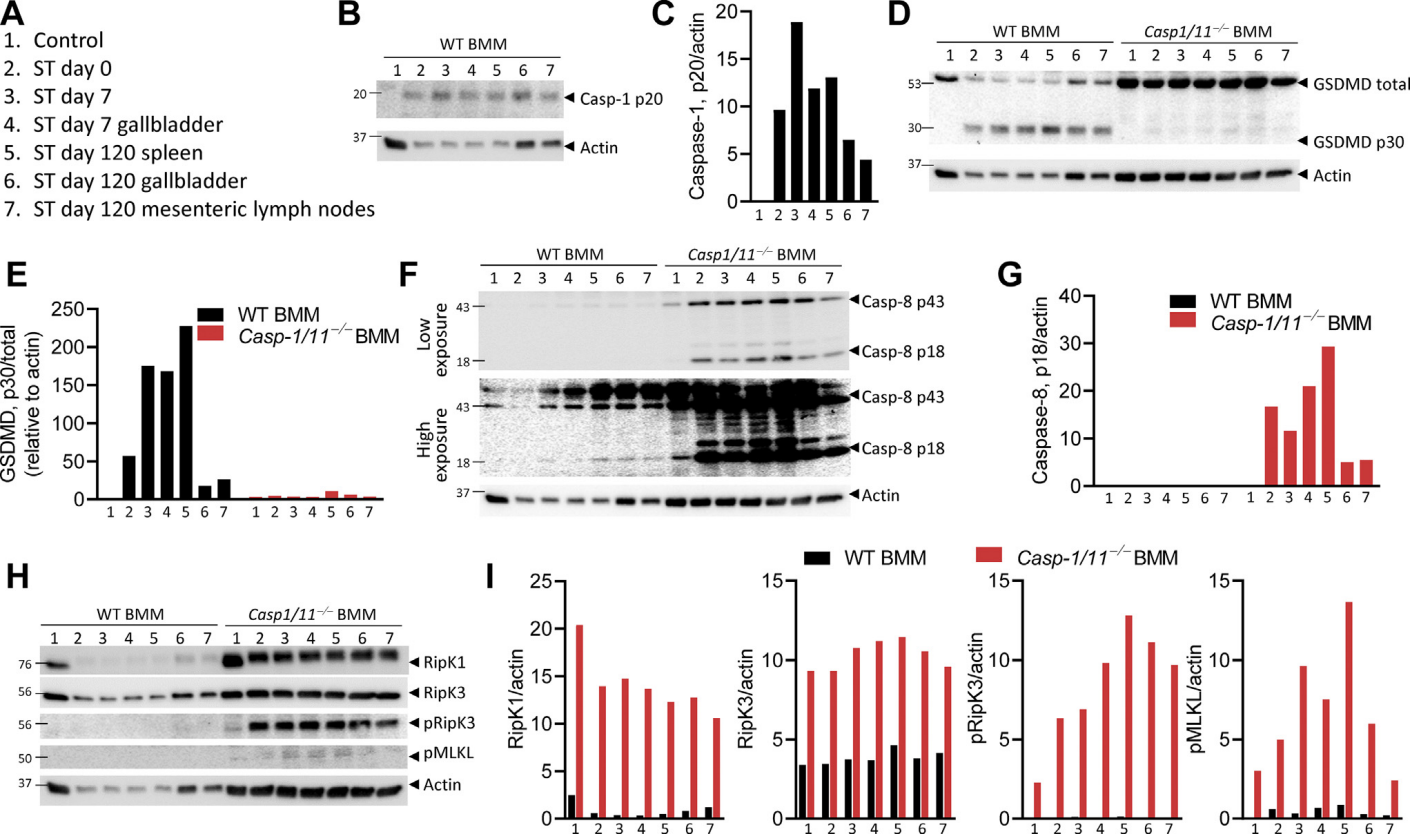
**Chronic ST isolates induce inflammasome independent cell death.** BMMs were differentiated from mice of wild-type or *caspase-1/11*^*−/−*^ backgrounds and infected with 10 MOI of the indicated bacterial isolates (*A*). Cell lysates were collected from the infected cells and Western blot analysis was performed to determine the expression of cleaved caspase-1, gasdermin D, cleaved caspase-8, RipK1, RipK3, pRipK3, pMLKL, and actin (*B*, *D*, *F* and *H*). Densitometric analysis was performed in Fiji to determine protein expression relative to actin (*C*, *E*, *G* and *I*). Blots in panels *B*, *D*, *F*, and *H* are from the same samples, hence the actin blots in these panels are the same. Results were repeated three times with BMMs generated from two mice/genotype in each experiment. BMM, bone-marrow-derived macrophage; MOI, multiplicities of infection.

Since the cell death of macrophages induced by ST-WT is dependent on Casp-1/11 (Fig. S1*A*), we sought to assess if the *Salmonella* isolates obtained from various organs displayed any modulation of their capacity to induce Casp-1/11-dependent cell death. We assessed the induction of Casp-1/11-dependent cell death by generating macrophages from WT and *Casp-1,11*^*−/−*^ mice and subsequently infecting the macrophages with the chronic *Salmonella* isolates. The extent of Casp-1/11-dependent cell death was progressively reduced in macrophages infected by chronic isolates obtained from the spleen and gallbladder (Fig. 3, *A* and *B*). Secretion of IL-1β remained dependent on Casp-1/11 (Fig. 3*C*). In contrast to NLRC4, the isolated bacteria displayed an impairment in their ability to induce NLRP3-dependent inflammasome signaling (Fig. 3, *D* and *E*). Activation of Casp-1 and GSDMD was also reduced in bacteria isolated at later time periods (Fig. 4, *A*–*E*). Interestingly, the mechanism of cell death did not switch toward apoptosis or necroptosis unless Casp-1/11 was disabled (Fig. 4, *F*–*I*). Thus, the processing of Casp-1/11 precludes the activation of other pathways of cell death.

### Flagellar apparatus promotes bacterial motility and induces cell death of macrophages, and this is modulated during chronic infection

**Figure 5:**
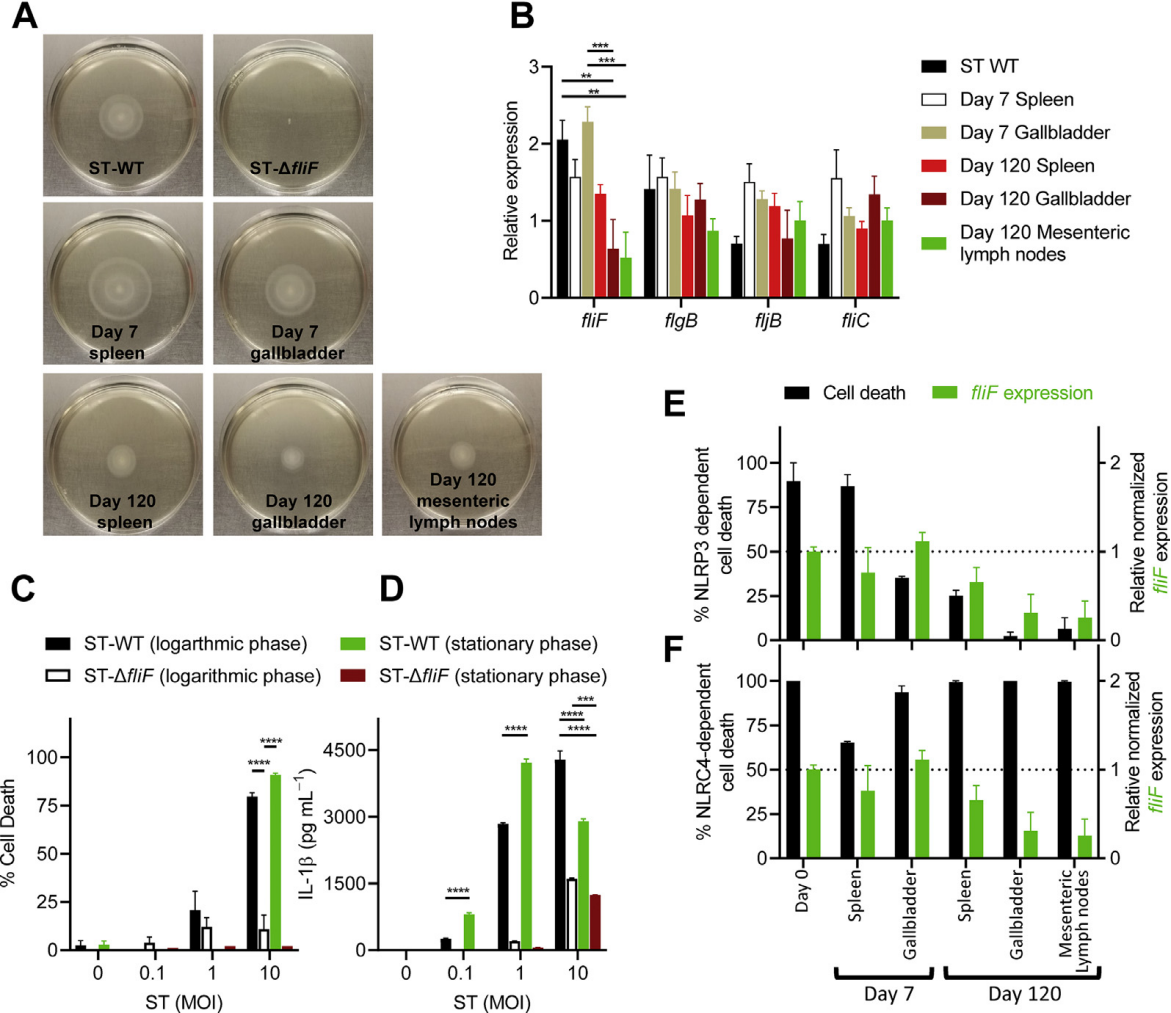
**Flagellar motility is modulated during chronic ST infection.** Swimming motility of bacteria was assessed after overnight incubation at room temperature (*A*). Relative gene expression was determined by quantitative RT-PCR for *fliF*, *flgB*, *fljB*, and *fliC* (*B*). BMMs were generated from wild-type C57BL/6J mice and pretreated overnight with 100 ng ml^−1^ LPS before infection with ST-WT or ST-Δ*fliF* cultured to logarithmic or stationary phase. After 3 h of infection, cell death was quantified by neutral red uptake, (*C*) and the secretion of IL-1β was measured by ELISA (*D*). NLRP3- and NLRC4- dependent induction of cell death by ST-WT or chronic ST isolates was compared against the expression of *fliF* (*E* and *F*). Values represent mean ± SEM. Results are representative of three separate experiments. Mean values were compared by two-way ANOVA with post-hoc Tukey’s multiple comparison test (∗∗*p* < 0.01; ∗∗∗*p* < 0.001; ∗∗∗∗*p* < 0.0001). BMMs were generated from two mice/genotype in each experiment. BMM, bone-marrow-derived macrophage; ST, *Salmonella typhimurium.*

We sought to assess if an alteration in flagellin expression was responsible for the modulation of cell death observed in our chronic *Salmonella* isolates. To characterize the motility of the *Salmonella* isolates, we performed swimming motility assays *in vitro*. In comparison to early isolates, chronic isolates exhibited reduced flagellar motility, as reflected by a reduction in the diameter of their swimming motility halo (Fig. 5*A*). In accordance with the reduced motility observed, a reduction in the expression of *fliF* was also observed by qRT-PCR in the chronic isolates (Fig. 5*B*). FliF forms the MS ring of the flagellar apparatus, and a reduction in the expression of this gene during chronic infection would negatively impact the assembly of the entire flagellar structure (24, 25, 26).

We also observed that ST deficient in *fliF* had an impaired ability to induce cell death and IL-1β secretion in infected macrophages (Fig. 5, *C* and *D*). Under SPI-I inducing conditions (logarithmic-phase culture), ST-Δ*fliF* induced low levels of cell death and moderate IL-1β secretion; however, it was significantly impaired in its ability to do so in comparison to ST-WT (Fig. 5*C*). In contrast, under SPI-II inducing conditions (stationary-phase culture), ST-Δ*fliF* was completely unable to induce cell death but retained its ability to induce IL-1β secretion (Fig. 5*D*). During the chronic stages of infection, NLRP3- but not NLRC4- dependent cell death correlated with the expression of *fliF* in ST isolated from various organs (Fig. 5, *E* and *F*). In logarithmic cultures of ST, inflammasome signaling was predominantly mediated by the NLRC4 inflammasome (Fig. S2, *A* and *B*) whereas in the stationary-phase cultures, inflammasome signaling showed increased dependence on NLRP3 (Fig. S2, *C* and *D*). Additional experiments indicated that the low-level inflammasome signaling in macrophages following infection by ST-Δ*fliF* was dependent on NLRP3 (Fig. S2, *E* and *F*). Since we observed that the cell death of macrophages was primarily mediated through Casp-1 signaling (Fig. S1), these results indicate that *fliF* is required for potent Casp-1 signaling in response to *Salmonella*.

### SPI-I is required for inflammasome signaling and is modulated during chronic infection

**Figure 6:**
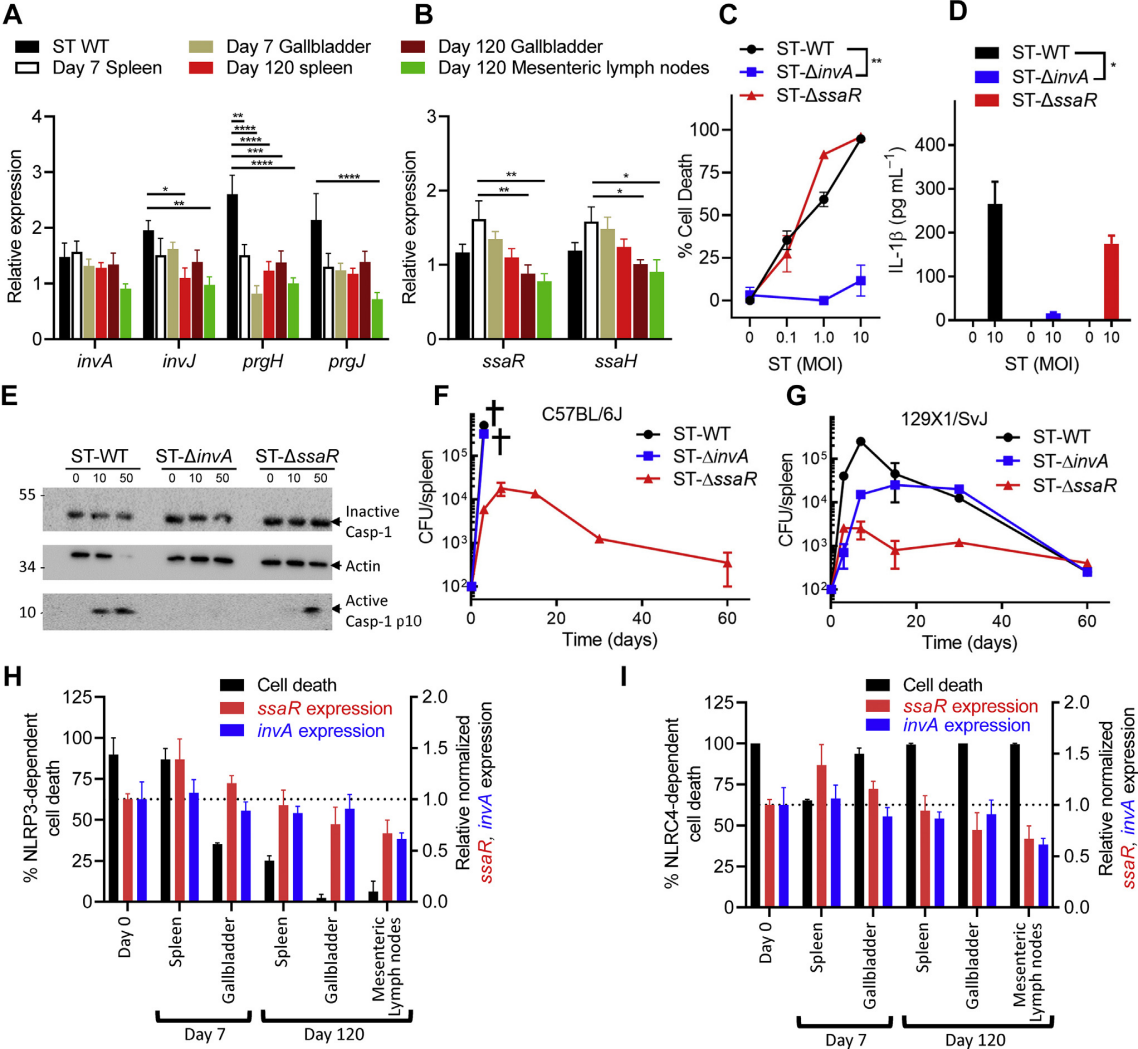
**ST-induced inflammasome signaling is dependent on SPI-I.** Relative gene expression was determined by quantitative RT-PCR for SPI-I genes *invA*, *invJ*, *prgH*, *prgJ* (*A*) and SPI-II genes *ssaR* and *ssaH* (*B*). BMMs were generated from wild-type C57BL/6J mice and infected with ST-WT, ST-Δ*invA*, or ST-Δ*ssaR* for 3 h. Cell death (*C*) and IL-1β (*D*) secretion were quantified by neutral red uptake assay and ELISA, respectively. Values represent mean ± SEM of triplicate cultures. Caspase-1 processing (*E*) was assessed by Western blotting. Spleens were harvested from C57BL/6J (*F*) and 129X1/SvJ (*G*) mice that were infected intravenously with 2 × 10^2^ CFU ST-WT, ST-Δ*invA*, or ST-Δ*ssaR*. Bacterial burden was evaluated at the indicated timepoints by plating serial dilutions on LB agar. NLRP3- and NLRC4-dependent induction of cell death by ST-WT or chronic ST isolates was compared against the expression of *ssaR* and *invA* (*H* and *I*). Mean values were compared by two-way ANOVA with post-hoc Tukey’s multiple comparison test (*A* and *B*) or Student’s *t* test (*C* and *D*) (∗*p* < 0.05; ∗∗*p* < 0.01; ∗∗∗*p* < 0.001; ∗∗∗∗*p* < 0.0001). Results are representative of three separate experiments and each experiment involved three mice per time point. SPI, *Salmonella* pathogenicity island; ST, *Salmonella typhimurium.*

We assessed whether the expressions of the SPI-I and SPI-II T3SSs were modulated during the course of chronic infection. To characterize the expression of these genes, we performed qRT-PCR for various SPI-I and SPI-II associated genes (Fig. 6, *A* and *B*). In comparison to ST-WT and early (Day 7) isolates, we observed that the expression of SPI-I and SPI-II genes was downregulated in chronic isolates. Notably, we observed that the expression of *prgJ*, a known NLRC4 activator (27), was significantly downregulated in chronic isolates. The dissemination and maintenance of *Salmonella* infection are dependent on the activity of its bacterial effectors secreted through its SPI-I and SPI-II T3SSs (28). The SPI-I T3SS secretes toxins, which promote bacterial infection in epithelial cells, whereas the SPI-II T3SS secretes toxins, which promote bacterial persistence in macrophages and epithelial cells (29, 30, 31, 32, 33, 34). We sought to assess the impact of the SPI-I and SPI-II secretion systems during bacterial infection by evaluating the survival of macrophages following infection by ST deficient in SPI-I (ST-Δ*invA*) or SPI-II (ST-Δ*ssaR*)-mediated secretion. Our results demonstrated that SPI-I mediated toxin secretion was indispensable for the induction of cell death, whereas SPI-II-mediated secretion was expendable (Fig. 6*C*). Similar results were observed for IL-1β secretion as SPI-I-deficient ST failed to induce IL-1β secretion in comparison to ST-WT or SPI-II-deficient ST (Fig. 6*D*). In addition, SPI-I-deficient ST (Δ*invA*) failed to readily induce Casp-1 activation (Fig. 6*E*) in comparison to ST-WT or SPI-II-deficient ST (Δ*ssaR*). These results demonstrate that the chronic isolates exhibit reduced expression of virulence factors including flagella and T3SS, which may be responsible for the reduced inflammasome signaling observed.

Although the SPI-I-deficient ST (Δ*invA*) failed to kill infected cells, it induced a lethal infection in C57BL/6J mice, like ST-WT, indicating that SPI-I does not influence host susceptibility in B6 mice (Fig. 6*F*). This is perhaps due to the mutation in *Nramp1* gene in C57BL/6J mice (22). In contrast, the mice remained resistant to infection by SPI-II deficient (ST-Δ*ssaR*) *Salmonella*, likely due to the bacteria’s inability to proliferate within phagocytes (Fig. 6*F*). In the resistant mouse strain 129X1/SvJ, SPI-I-deficient ST induced a chronic infection, like ST-WT, although the bacterial burden during the initial period was lesser with the SPI-I-deficient ST (Fig. 6*G*). At later time periods, the WT, SPI-I, or SPI-II-deficient ST induced a chronic low-level infection (Fig. 6*G*). These results demonstrate that the absence of functional SPI-I impaired the induction of macrophage cell death and impacted the burden of ST during the acute phase in 129X1/SvJ mice. However, SPI-I did not impact the chronicity of infection in the 129X1/SvJ strain of mice. We observed that the NLRP3-, but not NLRC4-, dependent cell death correlated with reduced expression of *ssaR* and *invA* (Fig. 6, *H* and *I*), although the correlation was not as strong as was observed with the *fliF* expression (Fig. 5, *E* and *F*).

### Inflammasome signaling offers a competitive advantage for ST only during the acute phase

**Figure 7:**
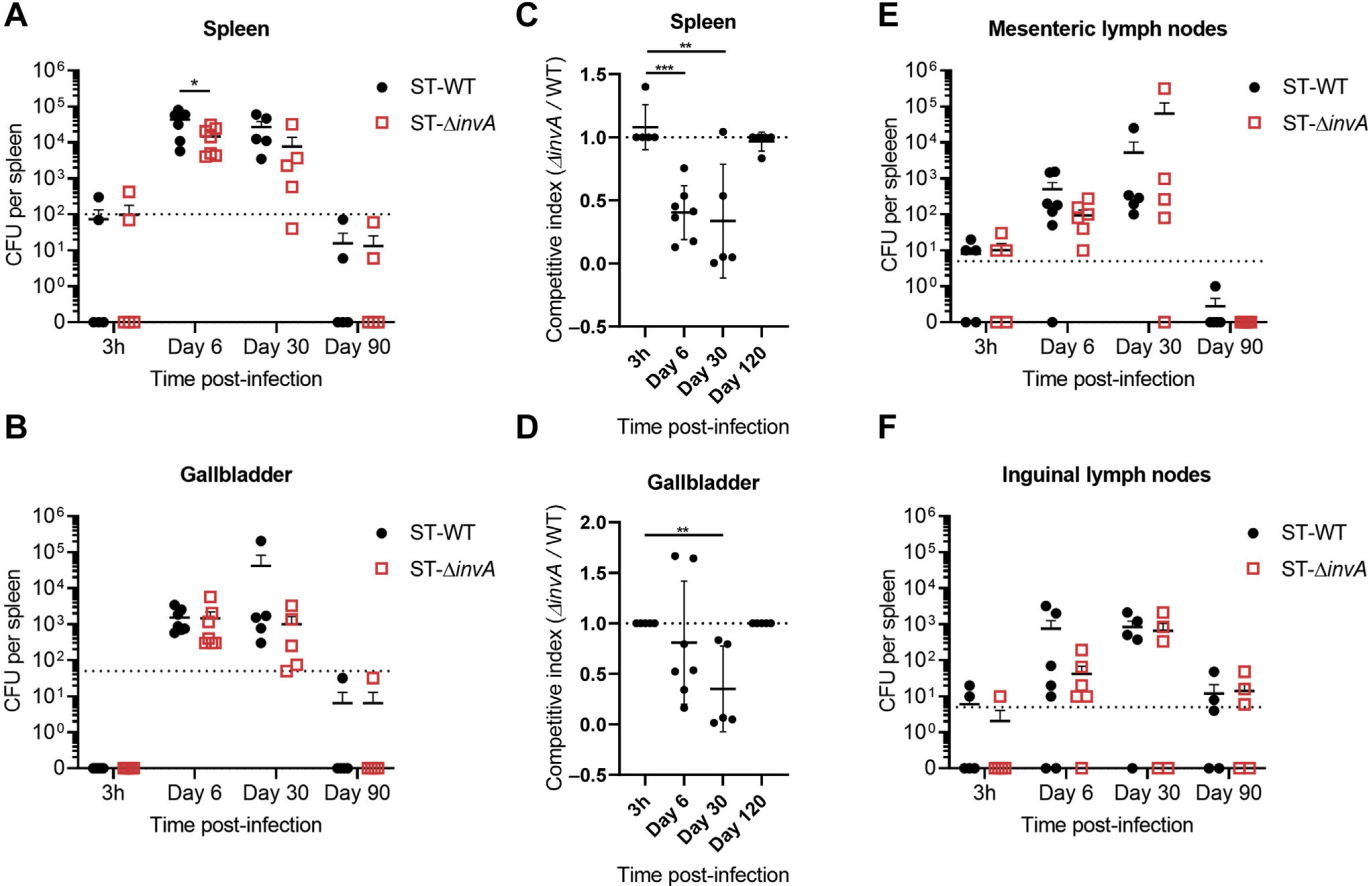
**SPI-I does not confer a competitive advantage to ST during chronic infection.** 129X1/SvJ mice were coinfected intravenously with an inoculum containing 1:1 CFU ST-WT and CFU ST-Δ*invA* (10^4^ CFU total) and sacrificed at the indicated timepoints. Bacterial burden in the spleen (*A*), gallbladder (*B*), mesenteric lymph nodes (*E*), and inguinal lymph nodes (*F*) was determined by plating serial dilutions of the homogenates on LB agar plates supplemented with 50 μg/ml of streptomycin or kanamycin to isolate ST-WT or ST-Δ*invA*, respectively. The competitive index was calculated from the ratio of ST-WT and ST-Δ*invA* present within the spleen (*C*) and gallbladder (*D*). A competitive index of 1 indicates an equal proportion of ST-WT and ST- Δ*invA*. Results represent the mean of five mice ±SEM at 3 h, day 30 and day 90 and mean of seven mice ±SEM at day 6. Results are representative of three experiments. Mean values were compared by Student’s *t* test (∗*p* < 0.05; ∗∗*p* < 0.01; ∗∗∗*p* < 0.001). SPI, *Salmonella* pathogenicity island; ST, *Salmonella typhimurium.*

We next sought to assess whether the presence of a functional SPI-I T3SS and consequent cell death of host cells conferred a fitness advantage to ST-WT. To address this, we coinfected 129X1/SvJ mice intravenously with an inoculum containing 1:1 ST-WT and ST-Δ*invA* (10^4^ CFU total) and sacrificed the mice at 6-, 30-, and 90-days post infection. We exploited the differential antibiotic sensitivity of ST-WT and ST-Δ*invA* to enumerate the relative proportion of the two bacteria in the same host. The spleen, gallbladder, mesenteric lymph nodes, and inguinal lymph nodes were excised and homogenized to release the intracellular bacteria. The homogenates were immediately plated on LB agar plates supplemented with 50 μg ml^−1^ of streptomycin or kanamycin to determine the relative number of ST-WT or ST-Δ*invA* present, respectively. The highest bacterial burden was observed at 6 d.p.i., and the detectable bacterial load tapered as the infection progressed. There was a reduction in bacterial counts of ST-Δ*invA* relative to ST-WT in the spleens at day 6 and 30 post infection (Fig. 7*A*). ST-Δ*invA* counts were also reduced in the gallbladder at day 30 post infection (Fig. 7*B*). We calculated the ratio of ST-WT *versus* ST-Δ*invA* in the same mouse and observed that the expression of SPI-I confers a survival advantage to ST during the initial periods of infection (Fig. 7, *C* and *D*). Interestingly, this survival advantage of ST-WT was lost at day 120. This result supports our previous findings since we have observed that the expression of SPI-I is reduced during the chronic period (Fig. 6*A*). Furthermore, the impact of SPI-I on fitness advantage appeared to be organ-specific (Fig. 7*F*).

### Chronic ST isolates exhibit reduced infectivity through the oral route

**Figure 8:**
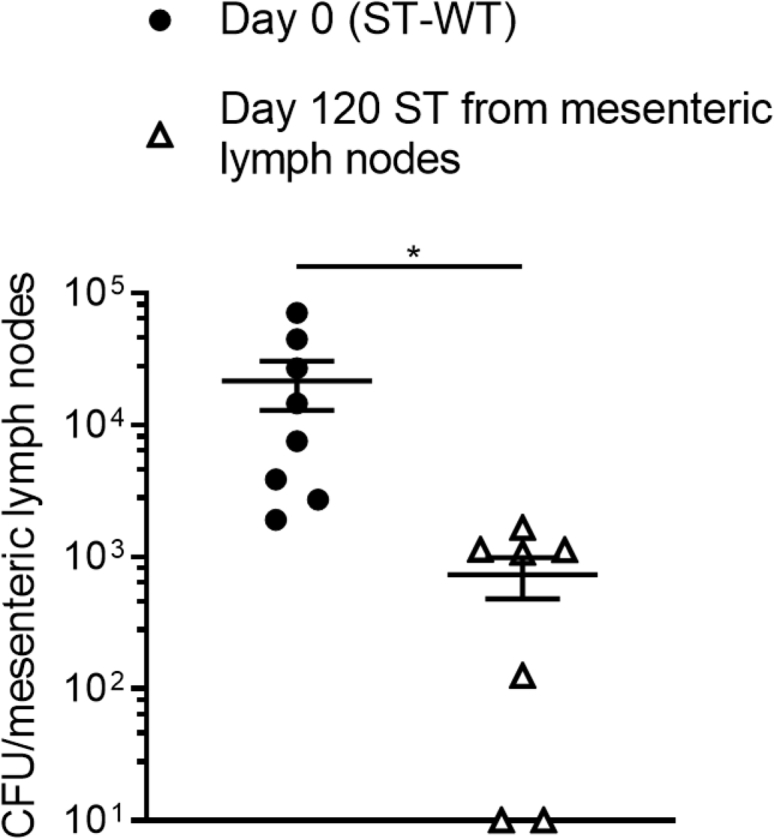
**Chronic ST isolates display attenuated virulence upon oral infection.** C57BL/6J mice were infected orally with 1 × 10^8^ CFU of ST-WT or the day 120 isolate from the mesenteric lymph node. Mice were sacrificed at 5 days postinfection, and the mesenteric lymph nodes were excised and homogenized. Serial dilutions of the homogenate were plated on LB agar to determine the bacterial burden. Results are representative of three experiments. Results represent the mean ± SEM of seven mice at each timepoint. Mean values were compared by Student’s *t* test (∗*p* < 0.05). LB, Luria–Bertani; ST, *Salmonella typhimurium*.

Our findings demonstrated that chronic isolates of ST exhibited reduced virulence in comparison to ST-WT in terms of inflammasome signaling, flagellar motility, and expression of various virulence genes implicated in flagellar assembly and SPI-I. We utilized a murine oral infection model to assess if the day 120 chronic isolate obtained from the mesenteric lymph node exhibited an impaired ability to infect hosts. In contrast to intravenous infection, which readily induces systemic infection, infection through the oral route is dependent on the SPI-I of ST since it promotes disruption of the gut epithelial barrier and dissemination of ST to the mesenteric lymph nodes and subsequently to the systemic compartments (35). Furthermore, infectivity of epithelial cells is also dependent on SPI-I (35, 36). We infected C57BL/6J mice orally with ST-WT or ST obtained from the mesenteric lymph node of mice at day 120 post infection. Mice were sacrificed at day 6 postinfection, and the mesenteric lymph nodes were excised and homogenized to release the intracellular bacteria. Serial dilutions of the homogenates were plated on LB agar plates supplemented with 50 μg ml^−1^ of streptomycin to quantify the bacterial burden. We observed reduced bacterial burden in mice infected by the isolate obtained at day 120 from mesenteric lymph nodes in comparison to mice infected by the ST-WT (day 0) (Fig. 8). These findings provide a functional confirmation of reduced SPI-I activity in the chronic ST isolate (day 120) obtained from the mesenteric lymph nodes.

## Discussion

Some bacterial pathogens can evade the host immune response and establish a chronic infection. Although the host can recognize the bacterial PAMPs and antigens to activate innate and adaptive immune responses against the chronic pathogen, these inflammatory responses are insufficient to clear the pathogen. Some pathogens evade recognition by T cells, whereas others modulate innate immune responses. Persistent activation of the inflammatory response at high levels during the course of a chronic infection would result in significant toxicity to the host. Thus, the host immune response must strike a balance between pathogen control and overt toxicity. In this study, we have used a mouse model of chronic *Salmonella* infection to decipher whether the bacterial isolates obtained from mice during the various stages of infection modulate host inflammasome signaling. We have shown that during the early stages of infection, inflammasome signaling is upregulated to facilitate pathogen control. However, as the pathogen persists for extended periods of time, inflammasome signaling is progressively tapered, although the ability to induce inflammasome signaling by itself does not influence the fitness of the bacterium.

Bacterial pathogens that localize intracellularly within the phagosome establish a chronic infection. *Mycobacterium tuberculosis* arrests phagolysosome maturation to create a niche permitting bacterial persistence and establishment of bacterial latency (37, 38). *Legionella pneumophila* is another bacterial pathogen that hijacks the host phagocytic pathway and inhibits phagosome maturation to create an intracellular environment favorable for bacterial replication (39). *Salmonella* resides within the phagosomes of infected cells where it utilizes its T3SS effectors to establish an intracellular niche conducive to bacterial replication (40). These secreted effectors function to maintain phagolysosome stability and inhibit further maturation of the *Salmonella* containing vacuole (29, 34, 41, 42). Persistent survival of *Salmonella* within the host is essential for the bacterium’s continued survival since it has no environmental reservoirs of infection (43, 44).

Pathogens that localize within the phagosome are not effectively cleared by the adaptive immune response since T cell priming is considerably delayed (45, 46, 47, 48), and once activated, the T-cell-mediated recognition of infected cells is poor due to a lack of rapid antigen presentation by infected cells (49). Loss of T cells results in a fatal infection (50, 51), which suggests that T cells control the infection, but fail to eradicate the phagosomal pathogens. Therefore, control of phagosomal pathogens such as *Salmonella* remains considerably dependent on the function of innate immune cells such as macrophages and neutrophils and the various innate immune signaling mechanisms employed by these cell types. Consequently, efforts to develop vaccines against phagosomal chronic pathogens such as *Salmonella* and *Mycobacterium* have been ineffective, despite the stable antigen expression exhibited by these pathogens (52, 53).

The protective role of inflammasome signaling against various pathogens such as *Listeria monocytogenes* and *Salmonella* has been reported (4, 7). In response to short-term infections, inflammasome activation elicits an acute inflammatory response toward the invading pathogen. After a brief period, the pathogen is eliminated, and the inflammatory response is resolved allowing the host to return to a normal homeostatic state. During chronic infections, the same inflammatory response is elicited; however, the infection fails to resolve, and the toxic proinflammatory cytokine response continues to be secreted in response to the ever-present pathogen. Over time, this continuous inflammatory response results in chronic inflammation and may lead to tissue toxicity if the inflammatory response persists at high levels. We have reported that in cystic fibrosis patients who harbor chronic infections, inflammasome signaling by the lung resident bacteria is considerably reduced (54).

Interestingly, we find that during the early period of infection, the inflammasome signaling ability of *Salmonella* is increased. This result corroborates the long-held practice of increasing virulence of pathogens by passing them in mice. SPI-I is a key virulence factor of *Salmonella* that promotes invasion into epithelial cells (34). However, the host uses inflammasome signaling to recognize SPI-I and control the pathogen by inducing death of infected cells and activation of the IL-1 cytokine family (18). If this process were to persist at high levels throughout the course of a chronic infection, it would be unfavorable for the host. Thus, an evolution toward reduced inflammasome signaling by the bacterium represents a strategy of mutual benefit. Since SPI-I and flagella are responsible for inflammasome signaling (55, 56), modulation of their activity by the bacterium during the chronic infection would result in reduction of inflammasome signaling. It has been reported that the transcription of virulence (flagellar) genes of ST is modulated in an organ-specific manner (57, 58). The selective pressure for the repression of bacterial virulence factors is also observed in other chronic bacterial infections, such as *Helicobacter pylori* in the gastric mucosa or *Pseudomonas aeruginosa* in the cystic fibrosis airway (54, 59, 60, 61). In these conditions, there is a selection for bacterial strains with mutations that repress or inactivate their virulence factors (62, 63, 64).

NLRP3 and NLRC4 are two of the key inflammasomes that are of importance during infection with *Salmonella* (18, 19, 20, 21). The NLRC4 inflammasome responds to the recognition of bacterial virulence factors including flagellin and the T3SS (27, 65, 66, 67, 68). On the other hand, the NLRP3 inflammasome signaling is more promiscuous in its activation by various sterile triggers such as oxidized cholesterol, urate crystals, amyloid aggregates, and a diverse array of cellular signaling including reactive oxygen species, K^+^ efflux, and Ca^2+^ mobilization, induced by host cellular damage (55, 56, 69, 70, 71, 72, 73, 74, 75). Recently it has been reported that *Salmonella* flagellin activates NLRP3 inflammasome signaling in human macrophages (76). Our results support this since we observed a reduction in NLRP3 signaling with chronic ST isolates and impaired inflammasome activation by ST-Δ*fliF*. NLRP3 has been shown to be important in *Salmonella* infection when the SPI-I activity is low (18, 77). In contrast, when SPI-I activity is high, NLRP3 has been demonstrated to possess a redundant role in the induction of cell death and IL-1β processing (78). Recognition by NLRC4/NLRP3 results in the assembly of the inflammasome complex, auto-proteolytic activation of caspase-1, and ultimately secretion of IL-1β. Inflammasome activation may also lead to inflammatory death of the infected cell through a process known as pyroptosis (79). In addition to cleaving pro-IL-1β, active caspase-1 may also cleave gasdermin D to initiate pyroptotic cell death (5). Cleaved gasdermin D translocates to the cell membrane where it forms a pore resulting in osmotic lysis of the cell (5, 80, 81, 82). Interestingly, we observed that under SPI-I inducing conditions, the NLRP3 inflammasome contributed moderately to the induction of cell death; however, its function was indispensable toward the secretion of IL-1β. In contrast, the function of the NLRC4 inflammasome was imperative for the induction of both cell death and IL-1β secretion. Under SPI-II inducing conditions, the NLRC4 inflammasome only contributed significantly toward IL-1β secretion, and its ablation did not significantly protect against the induction of cell death after infection. Furthermore, we observed that under SPI-II inducing conditions, the activity of the NLRP3 inflammasome contributed significantly toward the induction of both cell death and IL-1β secretion.

Bacterial flagellum is composed of structural, peripheral, filamentous, and cap proteins (24, 83). The basal MS ring is composed of 24 to 26 units of FliF, and it is the first flagellar structure to assemble (84). The MS ring serves as the mounting platform on which the flagellar apparatus assembles, and its presence is indispensable toward initiating flagellar assembly (85). FliC is a key filamentous protein that is exported to form the peripheral part of the filamentous structure (24). Both the NLRC4 and NLRP3 inflammasomes respond to FliC, resulting in inflammasome activation leading to IL-1β secretion and pyroptotic cell death (6, 76). Interestingly, FliC shares structural homology in the C-terminal region with PrgJ, a component of the SPI-I T3SS that also induces NLRC4 activation (27). Due to the conserved nature of the bacterial T3SS and flagella, it is possible for flagellar proteins such as FliC to be exported through the T3SS (19, 86). *Salmonella* FliC, a potent NLRC4 activator, may be translocated into host cells through the SPI-I T3SS (87). We observed that ST-Δ*fliF* could induce moderate levels of cell death and IL-1β secretion under SPI-I inducing conditions (logarithmic phase culture); however, ST-Δ*fliF* was unable to induce robust inflammasome activation in comparison to ST-WT. Furthermore, ST-Δ*fliF* grown under SPI-II inducing conditions (stationary phase culture) was completely unable to induce cell death but retained its ability to induce IL-1β secretion. The ability of stationary-phase ST-Δ*fliF* to induce IL-1β secretion was likely dependent on activation of the NLRP3 inflammasome by the SPI-II T3SS. ST SPI-II has been shown to induce NLRP3 activation (18), and NLRP3 ablation significantly protected against ST-induced IL-1β release upon infection. Together, these results demonstrate that both SPI-I secretion and flagellar expression are necessary to induce maximal inflammasome activation.

Although the cell death of macrophages induced by our lab adapted wild-type *Salmonella* was completely dependent on the activity of Casp-1/11, our results demonstrate that isolates of *Salmonella* obtained from infected mice are capable of inducing Casp-1/11-independent cell death. Previous studies have shown that *Salmonella* is capable of inducing Casp-1/11-independent death (88, 89, 90, 91). It is conceivable that *Salmonella* may switch toward the induction of noninflammatory types of cell death during the chronic stage to minimize tissue toxicity and promote host survival.

The acquired immune system is unable to mount an effective response against phagosomal pathogens such as *Salmonella* due to impaired antigen presentation (45, 46, 47, 48). Due to this impairment, the acquired immune system can suppress but not eradicate the infection. Thus, the innate immune system continues to play an important role in controlling bacterial proliferation within infected cells since its response is not dependent on effective antigen presentation. Although the innate response is not dependent on priming, our results demonstrate that its response also becomes compromised to favor bacterial survival through the selection of bacteria, which elicit a weaker innate immune response. The selection of bacteria, which do not actively express their virulence factors, leads to compromised inflammasome signaling over time. This progressive impairment favors bacterial survival since it allows the bacteria to circumvent immune recognition and proliferate within intracellular niches conducive to bacterial survival.

**Figure 9:**
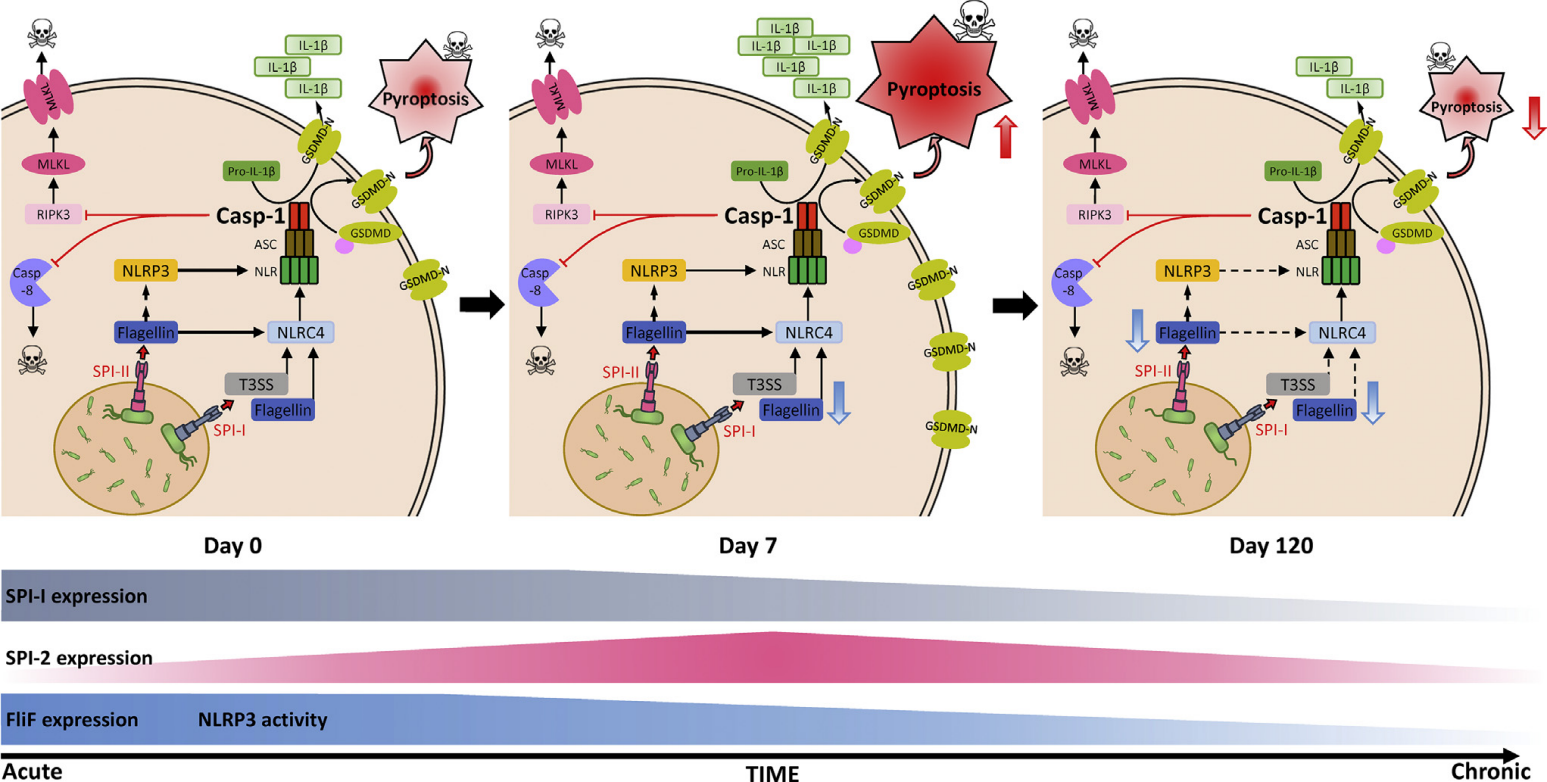
**Progressive modulation of cell death during the various stages of infection.** Early cell death is dominated by caspase-1, which limits the participation of other pathways in promoting cell death. The expression of SPI-I and FliF is initially high, which promotes inflammasome activation, and their expression progressively declines over the course of the infection. The expression of SPI-II genes is elevated during the acute phase and later declines during the chronic phase of infection. The expression of FliF undergoes the greatest decrease, which correlates with the progressive decrease in NLRP3-dependent cell death. NLRC4-dependent cell death is not affected since it can be activated by toxins/Flagellin secreted through SPI-I/II. SPI, *Salmonella* pathogenicity island.

Overall, we have revealed that inflammasome signaling is dynamically modulated during the chronic infection of mice with *Salmonella* (Fig. 9). The delicate balance of pathogen proliferation and host immune defenses forges a parity between the host and microbial interests to facilitate the cosurvival of both organisms. Pathogens that are highly virulent can kill hosts, whereas pathogens that possess relatively reduced virulence may persist. A key strategy for chronic survival of the virulent pathogen is to downregulate its ability to activate inflammasome signaling. Interestingly, we have revealed that the alternative pathways of cell death compensate for the reduction in inflammasome signaling to facilitate the pathogen control.

## Experimental procedures

### Mice

129X1/SvJ (JAX stock #000691), C57BL/6J (JAX stock #000664), and NLRP3-deficient (JAX stock #021302) mice were purchased from The Jackson Laboratory. Caspase-1,11-deficient mice were provided by Dr Richard Flavell (Yale University). B6.Nramp mice were obtained from Dr Greg Barton (University of California) and crossed with caspase-1, 11-deficeint mice to generate B6.Nramp.Casp-1,11-deficient mice. All mice were maintained at the University of Ottawa animal facility, and all animal experiments were performed at the University of Ottawa animal facility in accordance with the Canadian Council on Animal Care (CCAC) guidelines. All animal procedures were approved by the University of Ottawa Animal Care Committee.

### Bacteria and *in vivo* infection

Mice were infected with *Salmonella enterica* serovar Typhimurium (SL1344), ST-Δ*invA*, and ST-Δ*ssaR* strains from previously generated stocks stored at −80 °C. The bacteria were diluted in 0.9% NaCl, and the mice were injected intravenously *via* the lateral tail vein with the indicated dose of bacteria. Mice were sacrificed at the indicated timepoints, and the spleen, gallbladder, mesenteric lymph nodes, and inguinal lymph nodes were collected in cold PBS (Wisent Bio Products). The organs were homogenized to release the intracellular bacteria, and the bacterial burden was determined by plating serial dilutions of the homogenates on Luria–Bertani (LB) agar plates (BD Biosciences) supplemented with 50 μg ml^−1^ streptomycin (MilliporeSigma) or kanamycin (MilliporeSigma). Chronic isolates were generated by isolating single colonies, which were subsequently expanded in LB broth and frozen at −80 °C with 10% glycerol.

### Generation of murine bone-marrow-derived macrophages

Primary murine BMMs were generated from the bone marrow of C57BL/6J mice following a previously described procedure (92, 93). In brief, the mice were sacrificed, and bone marrow was harvested from the femur, tibia, and hip bones. The bone marrow cells were cultured in RPMI 1640 media (Gibco, Thermo-Fisher Scientific Inc) supplemented with 8% fetal bovine serum (Gibco), 50 μg ml^−1^ gentamicin (Gibco), and 5 ng ml^−1^ macrophage colony-stimulating factor (BioLegend). After 7 days, macrophages were harvested for usage.

### *In vitro* infection assays

For *in vitro* infections, BMMs were seeded in 96-well plates at a density of 10^5^ cells per well in RPMI 1640 media supplemented with 8% fetal bovine serum. Where indicated, cells were pretreated overnight with 100 ng ml^−1^ LPS (MilliporeSigma). The bacterial strains of interest were cultured to mid-exponential growth phase or stationary phase in LB broth. Bacterial density was approximated by adjusting the cultures to OD_600_ = 0.6 prior to infecting the cells at the indicated multiplicities of infection (MOI). Where indicated, cells were infected in the presence of MCC950 (Cayman Chemical Company), a NLRP3 inhibitor. The plates were subsequently centrifuged at 2500 rpm for 6 min to synchronize the bacterial uptake. Cells were infected for 30 min at 37 °C followed by gentamicin (50 μg ml^−1^) treatment to remove any extracellular bacteria. Cell viability was assessed at 3 h or 24 h postinfection as indicated.

### Cell death assay

Cell death was assessed by quantifying the uptake of neutral red, following a previously described procedure (90). In brief, cells were incubated with neutral red dye (MilliporeSigma) until viable cells became visibly red. The cells were then washed once with PBS to remove any free dye, and the cells were lysed with a solubilization solution to release the dye that had accumulated within live cells. The absorbance of the solubilized dye was quantified by colourimetric analysis at 570 nm on a FilterMax F5 microplate reader (Molecular Devices).

### Cytokine quantification

Cell culture supernatants were collected following the *in vitro* infections, and the expression of IL-1β was evaluated using an enzyme-linked immunosorbent assay (R&D Systems).

### Western blotting

Cell lysates were obtained by lysing cells in 1% SDS lysis buffer containing 1% β-mercaptoethanol (Gibco). The lysates were immediately boiled for 10 min to minimize protein degradation. Supernatants were diluted in SDS buffer and boiled for 10 min prior to loading. Western blot analysis was performed using the following antibodies: rabbit anti-cleaved caspase-1 (89332S; Cell Signaling Technology), rabbit anti-gasdermin D (39754S; Cell Signaling Technology), rabbit anti-mouse cleaved caspase-8 (8592P; Cell Signaling Technology), mouse anti-RipK1 (610459; BD Biosciences), rabbit anti-RipK3 (2283; ProSci Inc), rabbit anti-phospho RipK3 (91702S; Cell Signaling Technology), rabbit anti-phospho MLKL (37333S; Cell Signaling Technology), rabbit anti-caspase-1 p10 (SC-514; Santa Cruz Biotechnology), mouse anti-caspase-1 (sc-56036; Santa Cruz Biotechnology), mouse anti-β-actin (SC-81178; Santa Cruz Biotechnology), goat anti-mouse IgG HRP (172-1011; Bio-Rad Laboratories Inc), and goat anti-rabbit IgG HRP (A6154; MilliporeSigma). The blots were subsequently detected by chemiluminescence with SuperSignal West Pico PLUS Chemiluminescent Substrate (Thermo Fisher Scientific Inc) on a ChemiDoc MP imager (Bio-Rad Laboratories Inc).

### Motility assay

Swimming motility assays were performed following a previously described procedure (94). In brief, swim plates (LB broth with 0.3% w/v agar) were inoculated below the surface of the agar with a single colony of bacteria. The plates were incubated overnight at room temperature, and the diameter of the swimming motility halo was visualized.

### qRT-PCR

The bacterial strains of interest were grown to mid-exponential growth phase in LB broth, and total RNA was extracted using a Qiagen RNeasy kit, according to the manufacturer’s protocol (Qiagen). cDNA synthesis was performed using the iScript cDNA Synthesis Kit (Bio-Rad Laboratories Inc) according to the manufacturer’s instructions, and samples were stored at −20 °C until used. Quantitative real-time PCR was performed using the Bio-Rad CFX384 Touch Real-Time PCR System (Bio-Rad Laboratories Inc) in conjunction with SYBR Green PCR Master Mix (Applied Biosystems, Thermo Fisher Scientific Inc) using the following thermal cycling parameters: initial activation at 95 °C for 2 min, followed by 40 cycles of denaturation at 95 °C for 5 s, and annealing/extension at 60 °C for 30 s. On completion of the PCR amplification, a melt curve analysis was performed to confirm the presence of a single amplicon. Relative expression levels of the genes of interest were calculated using *dnaK*, *16s rRNA*, *rpoD*, and *rspM* as reference genes. Primers used for gene expression are listed in Table S1.

### Oral gavage infection

Mice were first gavaged with 20 mg streptomycin in distilled water the day before infection. The following day, mice were gavaged with the desired dose of bacteria in isotonic saline. Mice were sacrificed at the indicated timepoints, and the mesenteric lymph nodes were collected in cold PBS. The organs were homogenized to release the intracellular bacteria, and the bacterial burden was determined by plating serial dilutions of the homogenates on LB agar plates.

### Statistical analysis

Statistical analysis was performed using Prism 9 software (GraphPad Software). Student’s *t* test, one-way ANOVA with Tukey’s post-hoc test or two-way ANOVA with Tukey’s post-hoc test were used to confirm significance of results. *p* values <0.05 were considered statistically significant.

## Data availability

All the data are contained in this manuscript and the supplementary information.

## Supporting information

This article contains supporting information.

## Conflict of interest

The authors declare that they have no conflicts of interest with the contents of this article.
