# Supplementary material for: Isolates of Salmonella typhimurium circumvent NLRP3 inflammasome recognition in macrophages during the chronic phase of infection
Source: J Biol Chem. 2021 Dec 2;298(1):101461. doi: 10.1016/j.jbc.2021.101461 (PMC8715120; doi:10.1016/j.jbc.2021.101461)
Supplement: Supplemental Figures S1, S2 and Table S1 [file mmc1.pdf]

Supplementary table 1: Primer sequences for ST gene expression

fliC	FWD	GCAGTTAAGGTAGGCGATGAT
	REV	ACCGTCATCTGCAGTGTATTT
fliF	FWD	AAGCCATTCTGTCGCCTATC
	REV	GCTCTTCCGTCTGCTCTTTAT
flgB	FWD	CCGCAGTGGATCTGCTTTAT
	REV	TTGAGACTGTTATCCGCAAACT
fljB	FWD	TGACGCTACCGATGCTAATG
	REV	TCCTGTCGCTTCATCGTAATC
invA	FWD	AGCGTACTGGAAAGGGAAAG
	REV	CACCGAAATACCGCCAATAAAG
invJ	FWD	GGATGAGGTTGGCGGTTTAT
	REV	TACGAAAGCATCGCCATAGTC
prgH	FWD	ACAGCAGGCGTTACCTTATTC
	REV	AATTGACGGGCTCTGAGTATTT
prgJ	FWD	GGCGGTCAATATCAGGTCTATG
	REV	GGTCCTCAATCCTGTTGGTAAT
ssaR	FWD	CGTCATGGGAACTTCTTTCCT
	REV	AAGGCCATACAGTGCGATATT
ssaH	FWD	GCGTTAACCATAGCCTGATTTC
	REV	CCAACAATAATGCCAGACATACC
dnaK	FWD	CGCTTCCAGGACGAAGAAGT
	REV	GGCGCCATTTTCTGACCTTT
16s rRNA	FWD	AGATGGGATTAGCTTGTTGGTGA
	REV	GTAACGTCAATGCTGCGGTTA
rpoD	FWD	GGTCTGACCATCGAACAGGTG
	REV	ATCAGACCGATGTTGCCTTC
rspM	FWD	CGTTGGTCTTGGTACGCTGA
	REV	GTGATCTGCGCCGTGAAATC

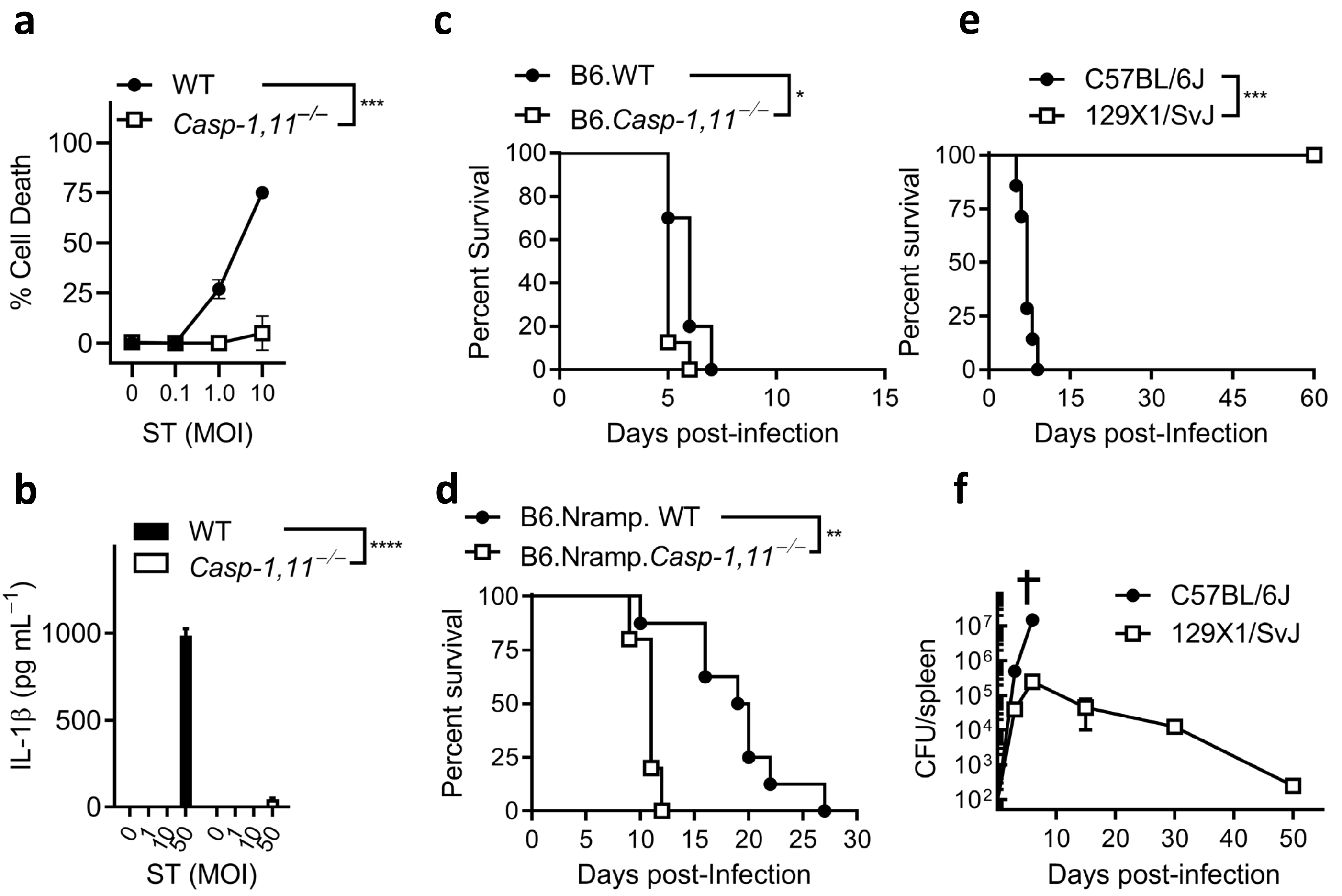

Supplementary Figure 1. *Salmonella Typhimurium* infection induces inflammasome activation. Bone marrow-derived macrophages (BMMs) were differentiated from mice of the indicated genotypes and infected with ST for 3 hours. Cell death (**a**) and IL-1β secretion (**b**) were quantified by neutral red uptake assay and ELISA, respectively. Mice of the indicated genotypes were infected intravenously with $2x10^2$ CFU ST and their survival (**c, d**) was assessed by monitoring the weight and clinical symptoms of the mice. C57BL/6J and 129X1/SvJ mice were infected intravenously with $2x10^2$ CFU ST and their survival was monitored (**e**). Mice were sacrificed at the indicated timepoints, and their spleens were excised to determine the bacterial burdens (**F**) by plating serial dilutions on LB agar plates. Mean values were compared by two-way ANOVA with post-hoc Tukey's multiple comparison test (**a, b**) or log-rank (Mantel-Cox) test (**c-e**) (*$P < 0.05$; **$P < 0.01$; ***$P < 0.001$; ****$P < 0.0001$). Each experiment was repeated three times. BMMs in panels **a** and **b** were generated from 2 mice from each genotype. Panels **c-e** contained 5 mice per group, and panel f contained 3 mice per group.

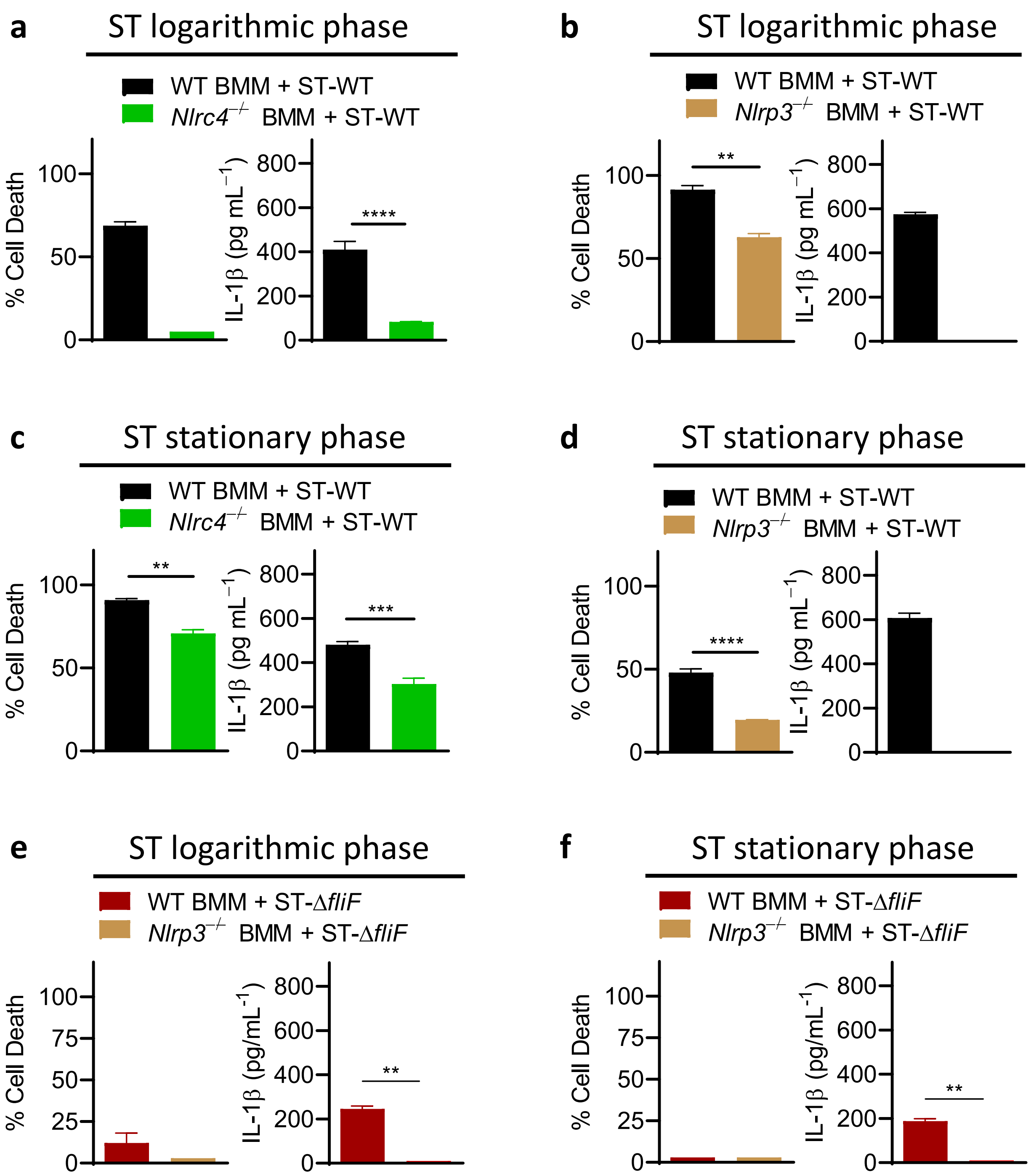

Supplementary Figure 2. ST induces NLRP3 dependent IL-1β secretion. Bone marrow-derived macrophages (BMMs) were differentiated from the bone marrow of the indicated genotypes and infected with 0.1 or 50 MOI of ST-WT or ST-Δ*fliF* cultured to logarithmic (a, b, e) or stationary (c, d, f) phases of growth, respectively. Cell death and IL-1β were quantified by neutral red uptake and ELISA, respectively. Values represent mean ± SEM. Mean values were compared by Student's *t*-test (*P < 0.05; **P < 0.01; ***P < 0.001; ****P < 0.0001). Each experiment was repeated three times. BMMs in panels **a-d** were generated from 2 mice from each genotype.
